# Cobalt Chloride Upregulates Impaired HIF-1α Expression to Restore Sevoflurane Post-conditioning-Dependent Myocardial Protection in Diabetic Rats

**DOI:** 10.3389/fphys.2017.00395

**Published:** 2017-06-13

**Authors:** Jianjiang Wu, Long Yang, Peng Xie, Jin Yu, Tian Yu, Haiying Wang, Yiliyaer Maimaitili, Jiang Wang, Haiping Ma, Yining Yang, Hong Zheng

**Affiliations:** ^1^Department of Anesthesiology, The First Affiliated Hospital of Xinjiang Medical UniversityUrumqi, China; ^2^Department of Anesthesiology and Guizhou Key Laboratory of Anesthesia and Organ Protection, Zunyi Medical CollegeZunyi, China; ^3^Department of Cardiology, The First Affiliated Hospital of Xinjiang Medical UniversityUrumqi, China

**Keywords:** diabetic state, ischemia-reperfusion, sevoflurane post-conditioning, myocardial protection, hypoxia inducible factor-1

## Abstract

Previous studies from our group have demonstrated that sevoflurane post-conditioning (SPC) protects against myocardial ischemia reperfusion injury via elevating the intranuclear expression of hypoxia inducible factor-1 alpha (HIF-1α). However, diabetic SPC is associated with decreased myocardial protection and disruption of the HIF-1 signaling pathway. Previous studies have demonstrated that cobalt chloride (CoCl_2_) can upregulate HIF-1α expression under diabetic conditions, but whether myocardial protection by SPC can be restored afterward remains unclear. We established a rat model of type 2 diabetes and a Langendorff isolated heart model of ischemia-reperfusion injury. Prior to reperfusion, 2.4% sevoflurane was used as a post-conditioning treatment. The diabetic rats were treated with CoCl_2_ 24 h before the experiment. At the end of reperfusion, tests were performed to assess myocardial function, infarct size, mitochondrial morphology, nitric oxide (NO), Mitochondrial reactive oxygen species (ROS), mitochondrial respiratory function and enzyme activity, HIF-1α, vascular endothelial growth factor (VEGF) and endothelial NO synthase (eNOS) protein levels. In addition, myocardial protection by SPC was monitored after the blood glucose levels were lowered by insulin. The diabetic state was associated with deficient SPC protection and decreased HIF-1α expression. After treating the diabetic rats with CoCl_2_, SPC significantly upregulated the expression of HIF-1α, VEGF and eNOS, which markedly improved cardiac function, NO, mitochondrial respiratory function, and enzyme activity and decreased the infarction areas and ROS. In addition, these effects were not influenced by blood glucose levels. This study proved that CoCl_2_activates the HIF-1α signaling pathway, which restores SPC-dependent myocardial protection under diabetic conditions, and the protective effects of SPC were independent of blood glucose levels.

## Introduction

Diabetes is an independent risk factor for perioperative complications and death associated with cardiovascular diseases, and the occurrence of myocardial ischemia is 1.45- to 2.99-fold higher in diabetic than in non-diabetic individuals (Whiting et al., 2011; Yeh et al., 2013). The diabetic state can interfere with the intrinsic protection mechanisms of ischemic preconditioning and post-conditioning during the reperfusion period, thereby increasing the myocardial infarction area (Ebel et al., 2003; Inamura et al., 2010).

Studies have demonstrated that sevoflurane post-conditioning (SPC) has protective effects on the myocardium of non-diabetic patients that resemble ischemic preconditioning, and this is a commonly used perioperative measure to avoid myocardial ischemia-reperfusion injury (Zhang et al., 2014; Cao et al., 2015; Yu et al., 2015). However, the protective effects of SPC are lost under diabetic conditions (Drenger et al., 2011; Tai et al., 2012), and the mechanisms are poorly understood. It has been reported that under diabetic conditions, the hypoxia-inducible factor-1alpha (HIF-1α) signaling pathway is damaged (Heather and Clarke, 2011; Xiao et al., 2013). Interestingly, previous data from our lab have suggested that the protective effects of SPC on the myocardium are due to an increase in the intranuclear expression of HIF-1α (Yang et al., 2016). Therefore, we examined whether the loss of SPC-induced myocardial protection under diabetic conditions was associated with defects in the HIF-1 signaling pathway.

HIF-1 is at the center of myocardial ischemia-reperfusion injury in healthy hearts and can augment the purine signaling pathway to facilitate myocardial protection (Eckle et al., 2008). In the diabetic state, the HIF-1 signaling pathway is compromised (Heather and Clarke, 2011), which reduces the abundance of some downstream components of this pathway such as the hypoxia-sensitive protein VEGF (Xiao et al., 2013). A previous study has revealed that cobalt chloride (CoCl_2_) can activate damaged HIF-1 under diabetic conditions, thereby protecting organs and decreasing diabetic nephropathy-related proteinuria and tubulointerstitial damage (Xi et al., 2004). In addition, CoCl_2_ treatment at low doses diminishes infarction size in non-diabetic rats via the upregulation and stabilization of HIF-lα expression (Nordquist et al., 2015). Nevertheless, it remains unknown whether CoCl_2_can restore the myocardial protective effects of SPC via upregulating HIF-1α expression under diabetic conditions.

To address these questions, we hypothesized that under diabetic conditions, the loss of the myocardial protective effects of SPC are associated with a compromised HIF-1α signaling pathway and that CoCl_2_ upregulates HIF-1α levels to restore the protective effects of SPC. In this study, an isolated heart model of ischemia-reperfusion injury was employed to investigate the influence of CoCl_2_treatment on the SPC-dependent protein expression of HIF-1α, VEGF, and eNOS in diabetic rats. We also examined whether such influences could improve respiratory function, enzymatic activity, NO, and the morphology of mitochondria as well as reduce the infarction area and ROS. In addition, the impact of insulin levels on SPC-dependent myocardial protection was examined.

## Materials and methods

### Establishment of a type 2 diabetic rat model

This study was approved by the First Affiliated Hospital of Xinjiang Medical University, Animal Ethics Committees (IACUC-20160218-032). Healthy male Sprague-Dawley (SD) rats (weighing 180–200 g) were obtained from the experimental animal center of the First Affiliated Hospital, Xinjiang Medical University, and the animals were maintained according to the Guide for the Care and Use of Laboratory Animals issued by the National Institutes of Health of the USA in 1996. The SD rats were fed a diet containing high levels of fat and sugar (57% normal diet, 18% lard, 20% sucrose, 2.5% cholesterol, and 2.5% egg yolk powder) for 4 weeks (Marfella et al., 2002; Ti et al., 2011). After fasting overnight, the animals were intraperitoneally injected with streptozotocin (STZ, Sigma, USA) at a dose of 40 mg/kg in 0.1 Mcitrate buffer (Sigma, USA). The modeling was considered successful if the blood glucose level was stabilized above 16.7 mmol/L (Skovso, 2014; He et al., 2015) 1 week after administration. These animals were used for the subsequent experiments.

### Drugs and reagents

Sevoflurane was purchased from Maruishi Pharmaceutical, Japan. Rabbit-anti-HIF-1α monoclonal antibody, rabbit-anti-VEGF monoclonal antibody, rabbit-anti-eNOS monoclonal antibody, and rabbit-anti-GAPDH monoclonal antibody were purchased from Sigma, USA. HIF-1α inhibitor 2ME2 was purchased from selleck, USA. Pentobarbital was purchased from Shanghai Tyrael Biological Technology Co., LTD.

### Experimental groups

**Figure 1:**
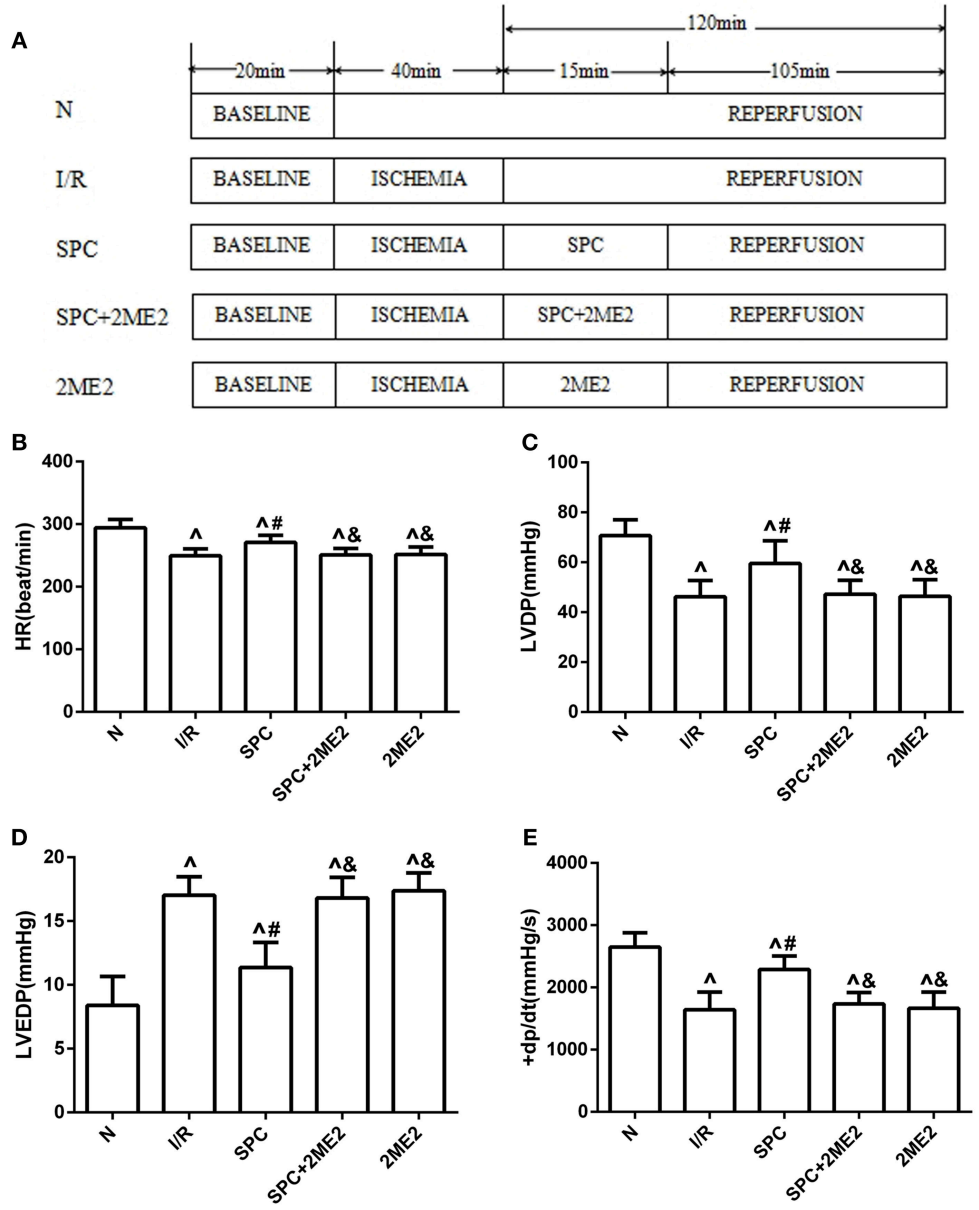
The schematic diagram of the experimental procedures. Except the N group, all hearts were subjected to 40 min whole heart ischemia, followed by reperfusion for 120 min. The SPC received 2.4% sevoflurane (1.0 MAC) treatment for 15 min, followed by reperfusion for 105 min. **(A)** The schematic diagram of non-diabetic rats. The cardiac function indicators under non-diabetic state. **(B)** Heart rate (HR, beat/per min). **(C)** Left ventricular developed pressure (LVDP, mmHg). **(D)** Left ventricular end-diastolic pressure (LVEDP, mmHg). **(E)** Maximum rate of increase of LV pressure (+dp/dtmax, mmHg/s; *n* = 12 /group). ^∧^*P* < 0.05 vs. N group; ^#^*P* < 0.05 vs. I/R group; and ^&^*P* < 0.01 vs. SPC group.

The diabetic rats along with their age-matched, healthy counterparts were randomly divided into the appropriate experimental groups. First, to examine whether SPC generates myocardial protection via upregulation of HIF-1α levels, the following groups were assembled: sham group (N); control group (ischemia-reperfusion group, I/R); SPC group (SPC); a specific HIF-1α inhibitor (2-methoxyestradiol, 2ME2) + SPC group (SPC + 2ME2); I/R + 2ME2 group (2ME2; Figure 1A).

The animals in the sham group were subjected to persistent perfusion of Krebs-Henseleit solution for 180 min. The animals in the I/R group were subjected to perfusion with 4°C St. Thomas cardioplegic solution after their hemodynamics had been balanced for 20 min. After 40 min of global ischemia at a temperature of 32°C, their hearts were subjected to reperfusion with Krebs-Henseleit solution for 120 min. The SPC group was subjected to perfusion with 4°C St. Thomas cardioplegic solution after their hemodynamics had been balanced for 20 min. After 40 min of global ischemia at a temperature of 32°C, their hearts were subjected to reperfusion with Krebs-Henseleit solution saturated with 2.4% sevoflurane (1.0 MAC) for 15 min, followed by reperfusion with standard Krebs-Henseleit solution for 105 min. The animals in the SPC + 2ME2 group were reperfused with Krebs-Henseleit solution supplemented with 2ME2 (2 μM) and saturated with 2.4% sevoflurane for 15 min, followed by reperfusion with standard Krebs-Henseleit solution for 105 min. The animals in the 2ME2 group were reperfused with Krebs-Henseleit solution supplemented with 2ME2 (2 μM) for 15 min, followed by reperfusion with standard Krebs-Henseleit solution for 105 min.

On the basis of these treatments, we further examined whether the SPC-dependent myocardial protection could be restored under diabetic conditions via CoCl_2_activation of HIF-1α using the following experimental groups: diabetic group (D); diabetic I/R group (I/R); diabetic SPC group (SPC); diabetic CoCl_2_ group (CoCl_2_); diabetic CoCl_2_+ SPC group (CoCl_2_+ SPC); diabetic CoCl_2_+ 2ME2 + SPC group (CoCl_2_ + 2ME2 + SPC); and diabetic CoCl_2_ + 2ME2 group (CoCl_2_ + 2ME2; **Figure 5A**).

Finally, to demonstrate the influence of normal insulin-mediated blood glucose levels on SPC-dependent myocardial protection, the following groups were used: diabetic SPC group (SPC); diabetic CoCl_2_+ SPC group (CoCl_2_ + SPC); diabetic + insulin + SPC group (SPC + Ins); and diabetic + insulin + CoCl_2_+ SPC group (CoCl_2_ + SPC + Ins; **Figure 9A**).

CoCl_2_ was intraperitoneally injected 24 h prior to cardiac ischemia at a dose of 30 mg/kg (Akinrinde et al., 2016). Blood glucose levels were controlled between 3.9 and 5.8 mmol/L with the following regimen: 3 U/d of intermediate-acting insulin was administered 48 h before cardiac ischemia, and 2U of short-acting insulin was administered 1 h before the experiment (Drenger et al., 2011). The Krebs-Henseleit solution was supplemented with the HIF-1α-specific inhibitor 2-methoxyestradiol 15 min prior to reperfusion (Si et al., 2014).

### Establishment of the langendorff reperfusion model (Bell et al., 2011)

Each rat was administered 500 U/kg of heparin intraperitoneally for anticoagulation, as well as 60 mg/kg of sodium pentobarbital for anesthesia, before the heart was immediately obtained and placed into 4°C Krebs-Henseleit solution (118 mmol/L NaCl, 4.7 mmol/L KCl, 1.2 mmol/L ${\text{MgSO}}_{4}^{.}$7H_2_O, 1.2 mmol/L KH_2_PO_4_, 25 mmol/L NaHCO_3_, 11 mmol/L glucose, 2.5 mmol/L CaCl_2_, pH 7.45) to drain the blood. The heart was then fixed onto a Langendorff perfusion needle, and retrograde aortic perfusion was performed using 37°C Krebs-Henseleit solution balanced with 95% O_2_–5% CO_2_. A homemade rubber balloon was inserted into the left ventricle via the left atrial appendage and mitral valve before the heart was connected to a Power Lab/8SP device. The perfusion pressure was maintained between 60 and 70 mmHg, and the size and position of the balloon were adjusted to keep the left ventricular end-diastolic pressure (LVEDP) between 0 and 10 mmHg. These steps were completed within 2 min. The inclusion criteria were as follows: 20 min after an isolated heart was balanced in 37°C Krebs-Henseleit solution for 20 min, the heart had a heart rate (HR) >250 beats/min and a left ventricular developed pressure (LVDP) >80 mmHg.

### Monitoring of hemodynamics

At the end of reperfusion, the Power Lab/8SP acquisition system was employed to record the following hemodynamic parameters: HR, LVDP, LVEDP, and maximum rate of increase of LV pressure (+dp/dt_max_, mmHg/s).

### Measurement of myocardial infarction area

At the end of reperfusion, the heart was immediately retrieved and placed into a −80°C freezer for 7 min. It was then sliced along the direction of apex-to-base into sections of 2–3 mm, which were incubated at 37°C in TTC solution (1%, pH 7.4) for 25 min. The slices were then fixed in 10% methanol overnight and photographed using a digital camera. The infarction area was quantified using the ImageJ software.

### Measurement of mitochondrial respiratory function

Myocardial mitochondria were extracted as described previously (Roussel et al., 2000). The mitochondrial protein content was quantified using a Bradford protein assay kit (Beyotime Biotechnology, China), and the protein concentrations of the samples were calculated based on the standard curve. Mitochondrial respiratory function was determined using oxygen electrodes (Hansatech) in a temperature-controlled oxygen meter (3 mL) with magnetic stirring. Briefly, the reaction solution was set to a total volume of 1,000 μL and a temperature of 37°C. Then, 900 μL of mitochondrial respiratory substrates were added before the solution was stirred and allowed to balance for 30 min. Next, 50 μL of mitochondrial suspension with a protein concentration of 1 mg/mL was added to the reaction solution, and the oxygen consumption curves were recorded for 20–30 s. After the oxygen curve stabilized, 1 μL of rotenone (final concentration 2 μM/L) and 10 μL of succinate (5 mM/L) were supplemented, whereby the mitochondria entered state IV respiration. The solution was then recorded for 1 min. After the oxygen consumption curve stabilized, 9 μL of ADP (100 μM/L) was added to the reaction solution, whereby the mitochondria entered state III respiration, and the oxygen consumption curve was recorded. Finally, once the ADP was completely phosphorylated to become ATP, the mitochondria again entered state IV respiration. The mitochondrial RCR = State III / State IV.

### Measurement of mitochondrial respiratory enzymatic activities

Oxygen electrodes (Hansatech) were used to measure mitochondrial respiratory enzyme activities as described previously (Schoepe et al., 2012). First, the mitochondria were thawed and frozen three times by shifting between 20 and −80°C to prepare the mitochondrial subunits. Next, the reaction solution (with a total volume of 1,000 μL) was supplemented with 900 μL of NADH-OX substrate, Cyt-OX substrate, and Suc-OX substrate and then incubated at 37°C for 15–20 min to reach a balanced state. Finally, 50 μL of the mitochondrial suspension with a protein concentration of 1 mg/mL was added to record the oxygen consumption curve for 5–10 min.

### Observation of the changes in myocardial mitochondrial morphology

After cardiac perfusion, the heart was retrieved, and the experimental materials were obtained from the isolated left ventricular wall using double-sided blades. The ventricular muscle was cut into cubes of 1 mm^3^, which were first fixed with 4°C glutaraldehyde phosphate buffer for 24 h before being subjected to dehydration, soaking, embedding, and staining. They were eventually cut into ultrathin sections of 50–70 nm, and the myocardial ultrastructure was observed under a transmission electron microscope.

### Western blotting

At the end of reperfusion, the myocardium in the ischemic risk zone was cut and immediately placed in liquid nitrogen. Total proteins were extracted from the myocardial tissue and lysed with tissue lysis buffer. The proteins in 30 μg of each sample were separated via SDS-PAGE, transferred onto a membrane, and blocked at 37°C for 2 h. HIF-1α, VEGF and endothelial NO synthase (eNOS) were probed with 1:1,000 dilutions of the respective antibodies overnight at 4°C. The membrane was then washed with TBST buffer and incubated with HRP-conjugated secondary antibodies (1:5,000) at room temperature for 1 h. Enhanced chemiluminescence images were analyzed using the Quantity One 2.6.2 software to determine the grayscale values for the target protein bands.

### Determination of nitric oxide (NO) content in myocardial tissue

After reperfusion, 100 mg of myocardial tissue was extracted and 0.9 mL of pre-cooled was added for preparation of tissue homogenate. The tissue homogenate was centrifuged at 10,000 g for 10 min at 4°C, and the supernatant was collected. The supernatant contained nitric oxide, nitrates, and nitrite. The Griess reagent was used to measure the nitrite content. The above method was used to determine the total nitrate and nitrite concentration and the total NO content was calculated.

### Mitochondrial reactive oxygen species (ROS) generation level assessment

The mitochondrial ROS generation level was determined by fluorometric method (Yang et al., 2016). We added mitochondria (0.5 mg) and mitochondrial ROS assay medium (2.9 mL) into a 3 mL quartz cuvette in one reaction system. In the other reaction system, 3 μL of 5 mmol/L 2′,7′-dichlorofluorescin diacetate (DCFH-DA) was added into 3.3 mmol/L succinic acid, as a substrate without mitochondria. We incubated the 2-reaction system at 37°C for 15 min, and then measured the fluorescence intensity of the reaction system with mitochondria (sample florescence intensity) and without mitochondria (basal fluorescence intensity). After that, we calculated the ROS generation rate by subtracting the basal fluorescence intensity from the sample florescence intensity.

### Statistical analyses

GraphPad Prism 6 software was used for the statistical analyses, and the measurement data are expressed as the mean ± standard deviation ($\bar{x}$ ± S). Analysis of variance in repeated measures was used for intra-group comparisons, whereas one-way ANOVA was used for inter-group comparisons. *P* < 0.05 was considered statistically significant.

## Results

### SPC upregulates HIF-1α to facilitate myocardial protection in a non-diabetic state

#### Cardiac function

At the end of reperfusion, the SPC group exhibited significantly higher HR, LVDP, and +dp/dt_max_ values than the I/R group, whereas LVEDP was significantly decreased (*P* < 0.05). However, the protective effects of SPC on the myocardium were abolished after administration of 2ME2 (*P* < 0.05, Figures 1B–E).

#### Myocardial infarction area

**Figure 2:**
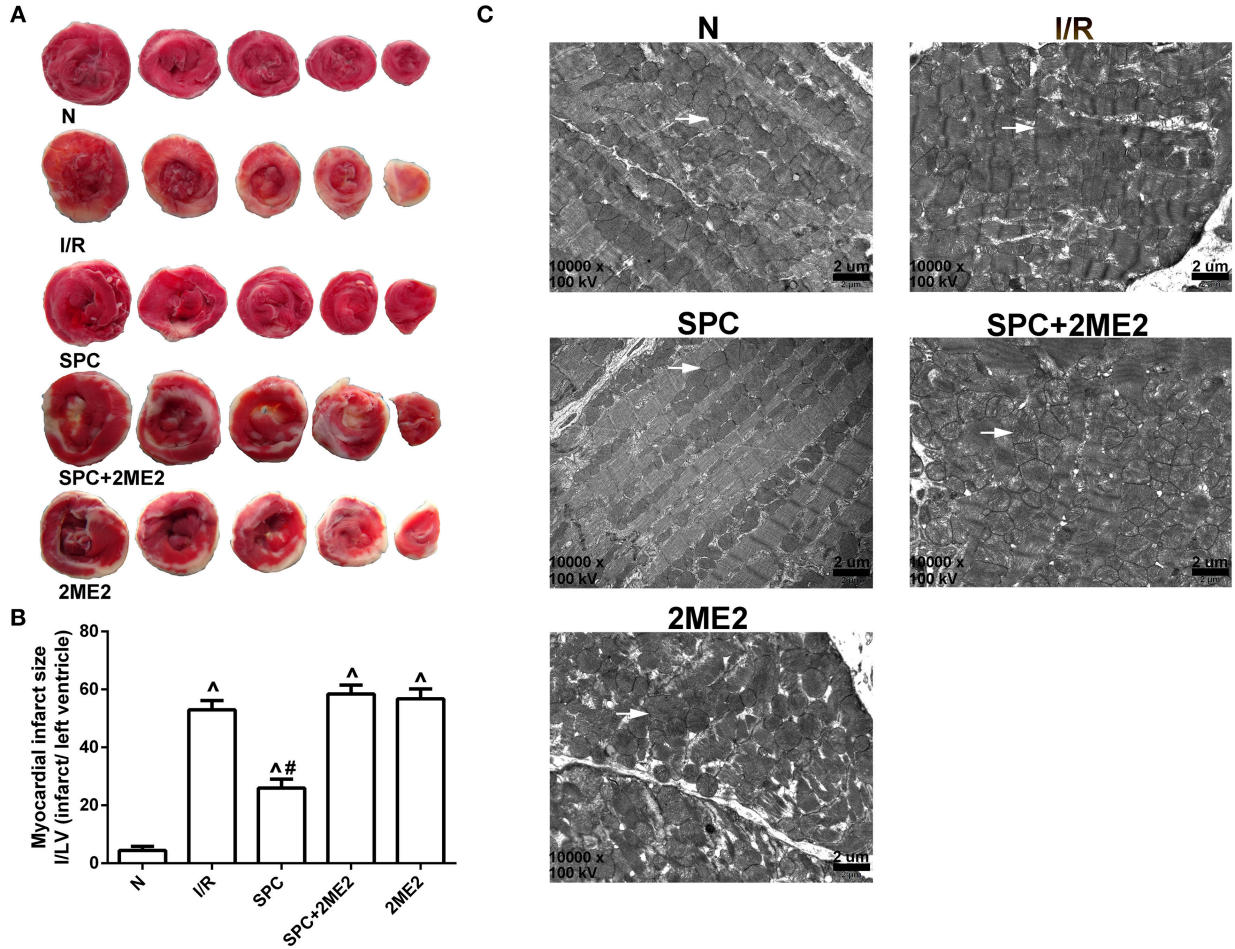
SPC reduced cardiac infarct size and improved mitochondrial ultrastructure under non-diabetic conditions. **(A)** Myocardial infarct area. **(B)** The infarct size was expressed as infarct/ left ventricle (I/LV; *n* = 5 /group). **(C)** Myocardial ultrastructure, white arrows indicate intact mitochondria and damaged mitochondria. ^∧^*P* < 0.05 vs. N group; ^#^*P* < 0.05 vs. I/R group.

Compared with the I/R group, SPC significantly decreased the myocardial infarction area (52.80 ± 3.19% vs. 25.60 ± 3.05%, *P* < 0.05). However, the infarction area increased by 58.20 ± 2.95% after 2ME2 administration (Figures 2A,B).

#### Mitochondrial ultrastructure

The mitochondria of the sham group were intact. However, the I/R group exhibited severely impaired myocardial structure with dissolved and severed myofilaments and prominent mitochondrial swelling, which was evidenced by a widened, ruptured ridge and membrane gaps with excessive expansion of sarcoplasmic reticulum. The myocardial texture of the SPC group was clear, and the myofilament arrangement was well organized, but some of the myofilaments and sarcomere spaces were widened and dissolved. Most of the mitochondria had intact morphology, and there were many with ridged membranes that were clearly visible and not dissolved or ruptured, although some minor swelling was observed. The SPC+2ME2 and 2ME2 groups exhibited mitochondrial injuries that were similar to those of the I/R group (Figure 2C).

#### Mitochondrial respiratory function and enzyme activity

**Figure 3:**
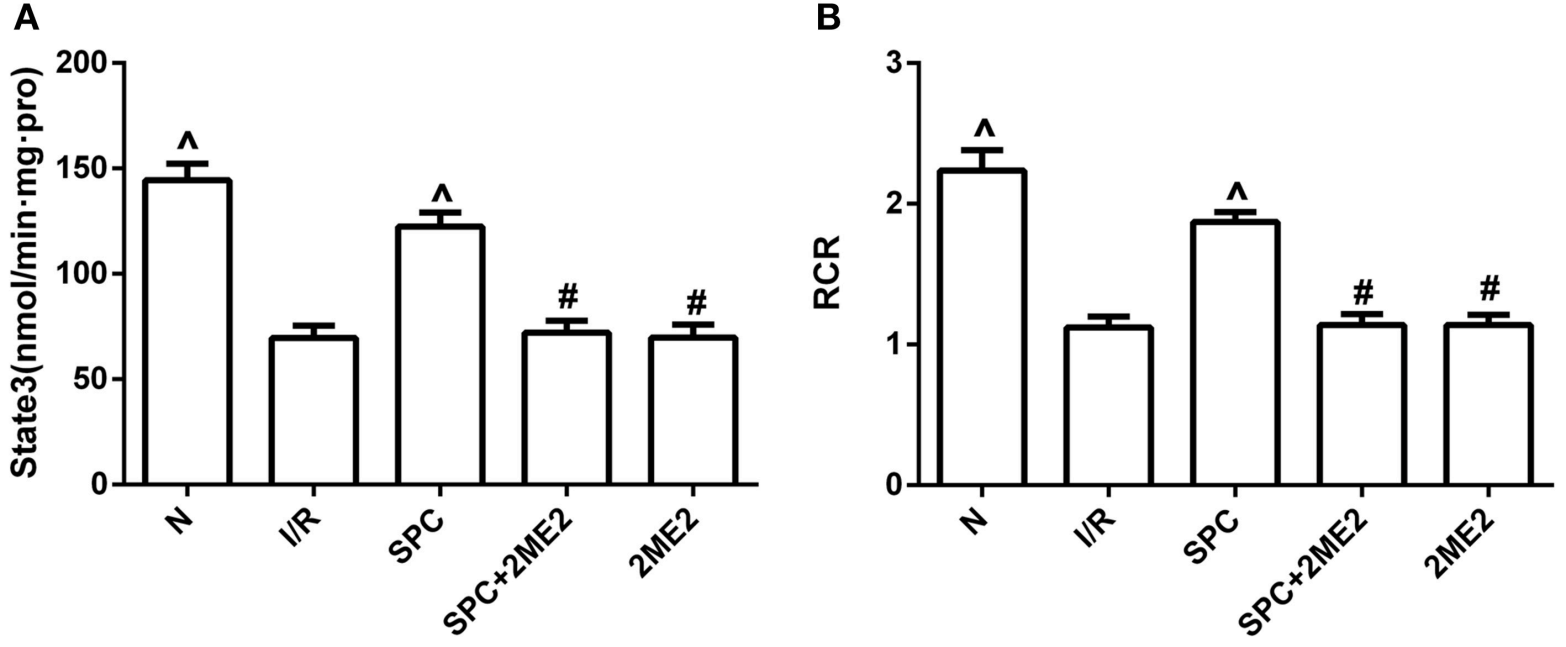
SPC improved mitochondrial respiratory function under non-diabetic conditions. **(A)** Mitochondrial state III respiration. **(B)** Mitochondrial respiratory control rate (RCR; *n* = 12 /group). ^∧^*P* < 0.05 vs. I/R group; ^#^*P* < 0.05 vs. SPC group.

Mitochondrial state III respiration, the respiration control rate (RCR), and the activities of NADH-OX, Cyt-OX, and Suc-OX were markedly improved in the SPC group. However, the protective effects of SPC on mitochondrial respiration and enzymatic activities were reversed by administration of 2ME2 (*P* < 0.05, Figures 3A,B, Table 1).

**Table 1:** The changes in mitochondrial respiratory enzymatic activities under non-diabetic conditions.

	**NADH-OX**	**Cytc-OX**	**SUC-OX**
N	285.90 ± 13.38^∧^	81.64 ± 6.14^∧^	81.50 ± 6.00^∧^
I/R	204.07 ± 11.70	48.38 ± 4.43	46.96 ± 3.71
SPC	252.95 ± 13.21^∧^	69.05 ± 4.98^∧^	66.98 ± 5.32^∧^
SPC + 2ME2	209.55 ± 9.61^#^	50.42 ± 5.65^#^	48.85 ± 4.22^#^
2ME2	209.03 ± 10.06^#^	49.37 ± 5.84^#^	48.75 ± 3.63^#^

*Data are presented as the mean ± SEM (n = 6). NADH-OX: NADH oxidase; Cytc-OX: Cytochrome C oxidase; SUC-OX: succinate oxidase; N: normal; I/R: ischemia reperfusion; SPC: sevoflurane post-conditioning; 2ME2: 2-Methoxyestradiol*.

∧ *P < 0.05 vs. I/R group*;

# *P < 0.05 vs. SPC group*.

#### Expression of the HIF-1α, VEGF and eNOS proteins

**Figure 4:**
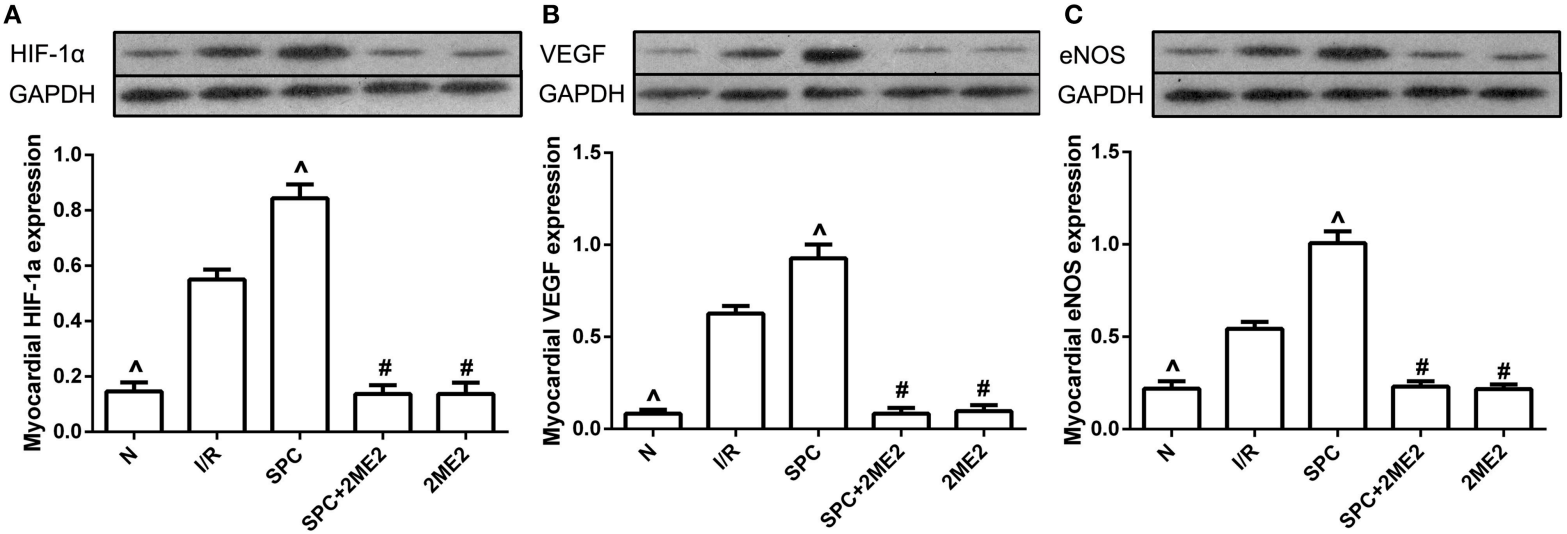
SPC upregulated the HIF-1α and VEGF expression under non-diabetic condition. **(A)** HIF-1α expression. **(B)** VEGF expression. **(C)** eNOS expression (*n* = 3 /group). ^∧^*P* < 0.05 vs. I/R group; ^#^*P* < 0.05 vs. SPC group.

At the end of reperfusion, the SPC group exhibited clearly elevated protein levels of HIF-1α and its downstream mediator VEGF and eNOS. However, this increase was abolished by 2ME2 treatment (*P* < 0.05, Figures 4A–C).

#### NO content in myocardial tissue

The NO content of myocardial tissue in the SPC group after reperfusion was significantly increased (*P* < 0.05, vs. I/R group). However, 2ME2 abolished the above-mentioned effect of SPC (*P* < 0.05, Table 2).

**Table 2:** The changes in myocardial NO and mitochondrial ROS under non-diabetic conditions.

	**ROS**	**NO**
N	5.32 ± 0.13^∧^	1.22 ± 0.08^∧^
I/R	11.46 ± 0.15	2.21 ± 0.12
SPC	8.13 ± 0.17^∧^	3.20 ± 0.13^∧^
SPC + 2ME2	11.80 ± 0.12^#^	1.97 ± 0.10^#^
2ME2	11.86 ± 0.15^#^	2.02 ± 0.07^#^

*Data are presented as the mean ± SEM (n = 6). ROS: Reactive oxygen species; NO: Nitric oxide; N: normal; I/R: ischemia reperfusion; SPC: sevoflurane post-conditioning; 2ME2: 2-Methoxyestradiol*.

∧ *P < 0.05 vs. I/R group*;

# *P < 0.05 vs. SPC group*.

#### ROS level in mitochondria

SPC significantly inhibited the mitochondrial ROS level after I/R (*P* < 0.05, vs. I/R group), but 2ME2 abolished this effect of SPC (*P* < 0.05, Table 2).

### Under diabetic state, impaired HIF-1α expression weakens SPC-dependent myocardial protection, which is restored by the CoCl_2_-induced activation of HIF-1α

#### Cardiac function

**Figure 5:**
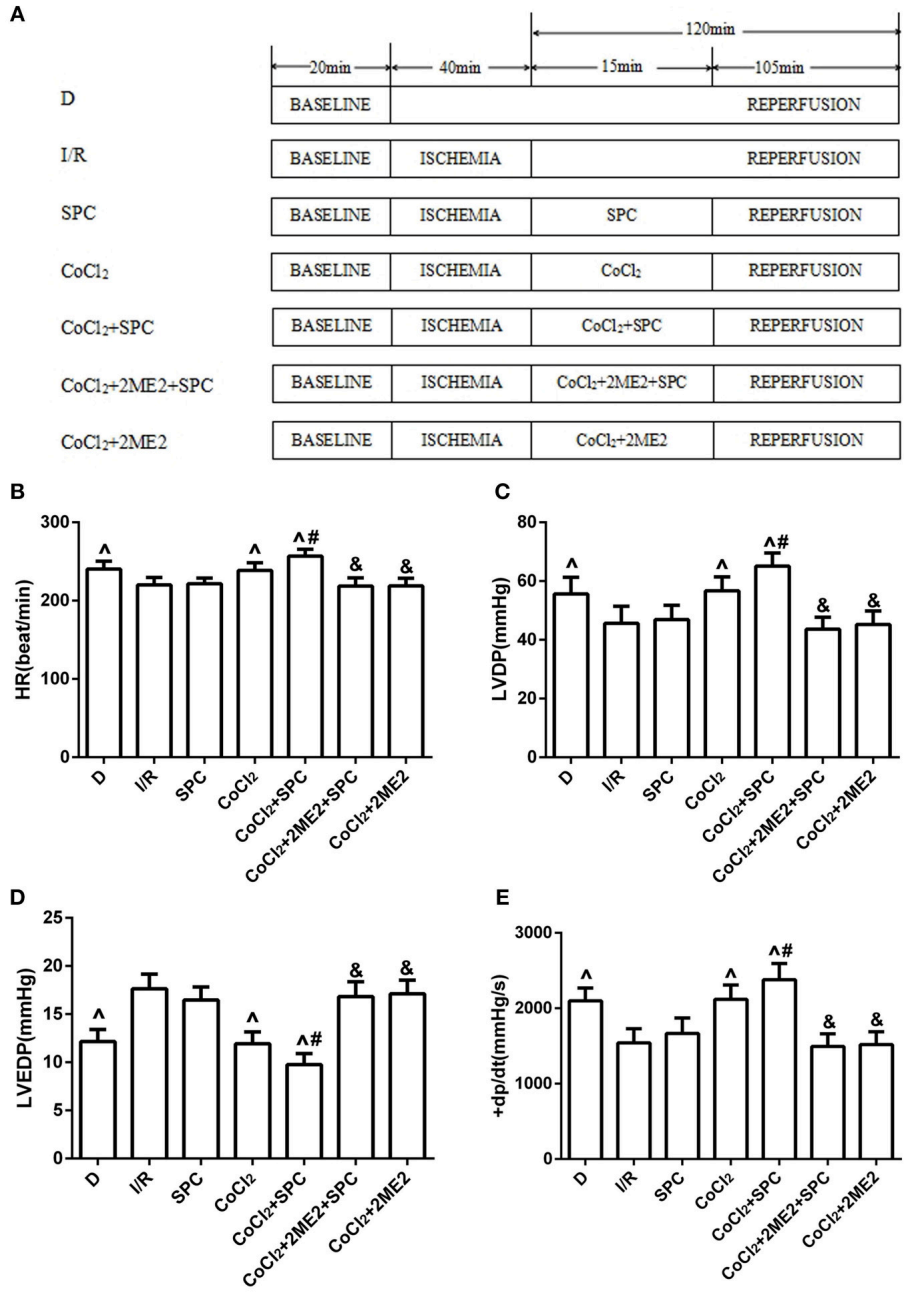
The schematic diagram of the experimental procedures. Except the D group, all hearts were subjected to 40 min whole heart ischemia, followed by reperfusion for 120 min. The SPC received 2.4% sevoflurane (1.0 MAC) treatment for 15 min, followed by reperfusion for 105 min. **(A)** The schematic diagram of diabetic rats. SPC improved the cardiac function indicators after CoCl_2_treatment. **(B)** Heart rate. **(C)** Left ventricular developed pressure. **(D)** Left ventricular end-diastolic pressure. **(E)** Maximum rate of increase of LV pressure (*n* = 10 /group). ^∧^*P* < 0.05 vs. I/R group; ^#^*P* < 0.05 vs. CoCl_2_ group; and ^&^*P* < 0.01 vs. CoCl_2_ + SPC group.

There were no significant differences in cardiac function between the SPC group and the I/R group (*P* > 0.05). After CoCl_2_treatment, there was a significant decrease in LVEDP, and the HR, LVDP, and +dp/dt_max_ values were increased in the CoCl_2_ + SPC group (*P* < 0.05 vs. the SPC group or I/R group). However, this was abolished by administration of 2ME2 (*P* < 0.05, Figures 5B–E).

#### Myocardial infarction area

**Figure 6:**
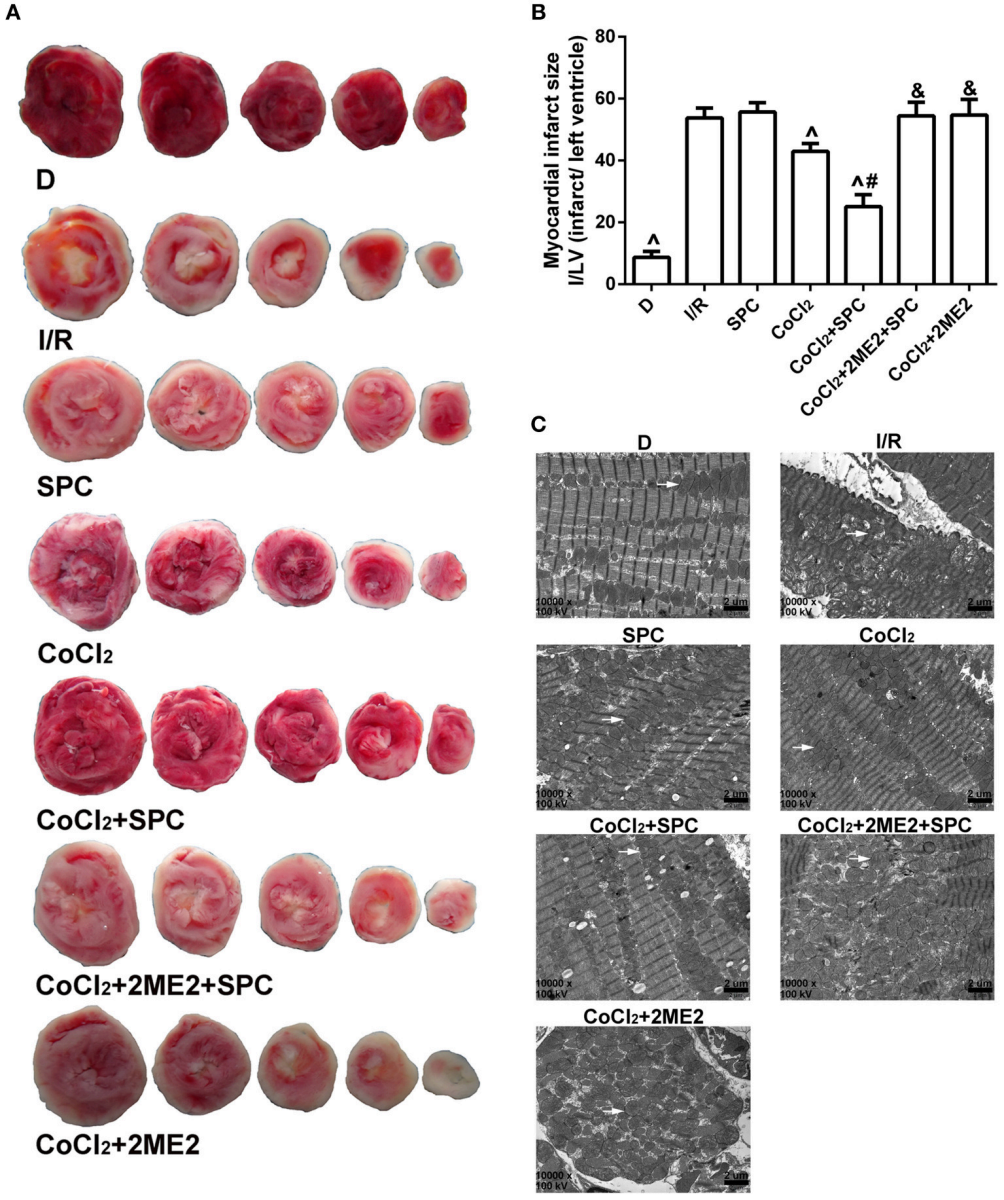
Under diabetic conditions, SPC reduced cardiac infarct size, and improved mitochondrial ultrastructure after CoCl_2_treatment. **(A)** Myocardial infarct area. **(B)** The infarct size was expressed as infarct/ left ventricle (I/L; *n* = 5/group). **(C)** Myocardial ultrastructure, white arrows indicate intact mitochondria and damaged mitochondria. ^∧^*P* < 0.05 vs. I/R group; ^#^*P* < 0.05 vs. CoCl_2_ group; and ^&^*P* < 0.01 vs. CoCl_2_ + SPC group.

There were no appreciable differences in infarction area between the I/R group and the SPC group (*P* > 0.05). However, the infarction size of the CoCl_2_ group was significantly decreased compared with that of the SPC group (42.99 ± 1.79% vs. 55.71 ± 3.00%, *P* < 0.05). In addition, the infarction area of the CoCl_2_ + SPC group was significantly smaller than that of the CoCl_2_ group (25.08 ± 3.87%, *P* < 0.05). Nonetheless, the infarction area was increased by 54.45 ± 4.40% after administration of 2ME2 compared with the SPC + CoCl_2_group (*P* < 0.05, Figures 6A,B).

#### Mitochondrial ultrastructure

The mitochondrial structure of the D group was intact. The myofilaments of the I/R group were dissolved or even severed, and prominent mitochondrial swelling was observed, which was evidenced by widened and ruptured ridges and membrane gaps, as well as excessive expansion of the sarcoplasmic reticulum. The SPC group exhibited comparable results to the I/R group. Although, the CoCl_2_ group had less severe mitochondrial impairment than the SPC group, it still manifested some minor defects such as mitochondrial swelling and irregular arrangement, as well as partially dissolved myofilaments. The mitochondria of the CoCl_2_ + SPC group appeared to be intact with an orderly arrangement, but some swelling was still observed. Nevertheless, there were apparent impairments such as severely damaged mitochondrial structure, dissolved myofilaments, and apparent swelling after administration of 2ME2 (Figure 6C).

#### Mitochondrial respiratory function and enzyme activity

**Figure 7:**
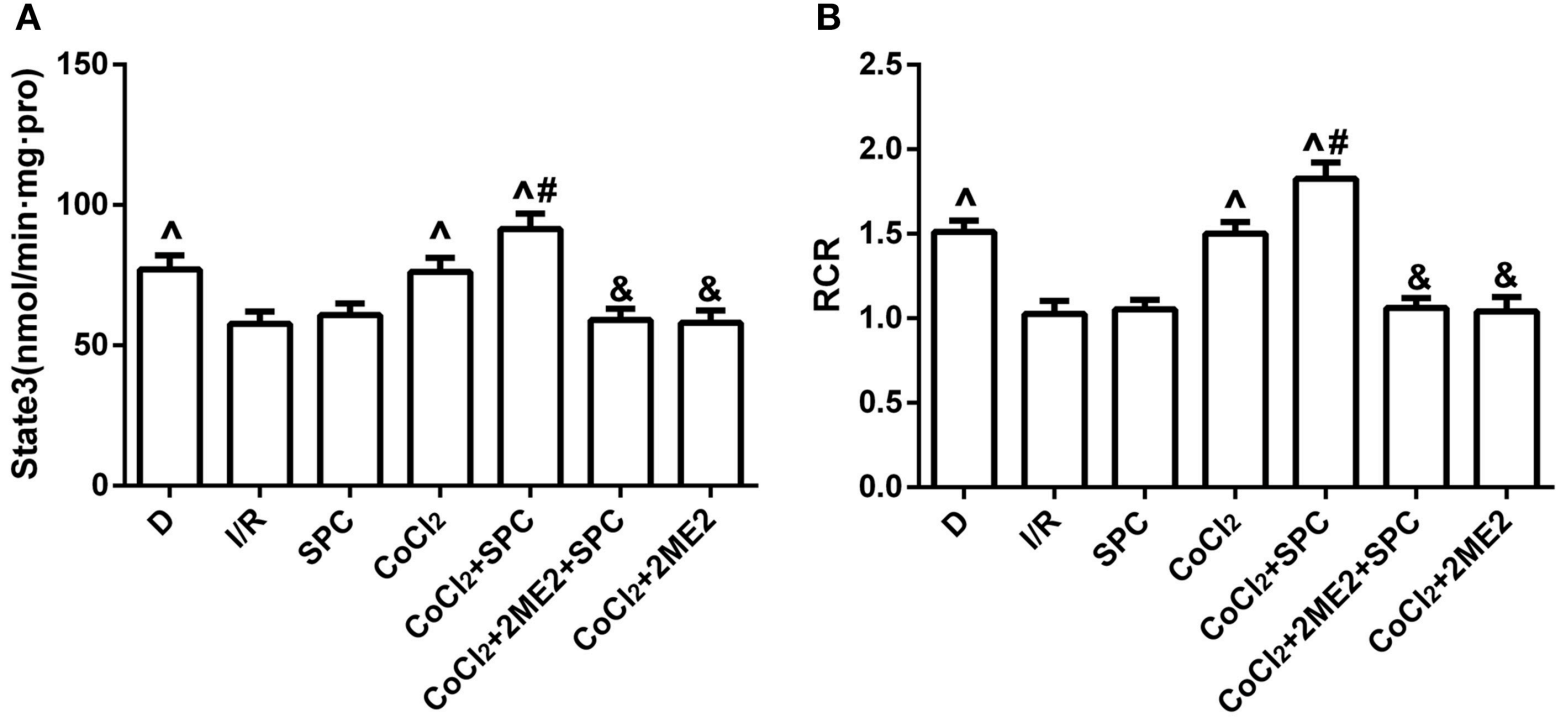
Under diabetic state, SPC improved the mitochondrial respiratory function after CoCl_2_treatment. **(A)** Mitochondrial state III respiration. **(B)** Mitochondrial respiratory control rate (RCR; *n* = 10 /group). ^∧^*P* < 0.05 vs. I/R group; ^#^*P* < 0.05 vs. CoCl_2_ group; and ^&^*P* < 0.01 vs. CoCl_2_ + SPC group.

There were no significant differences in state III respiration and RCR between the SPC group and the I/R group (*P* > 0.05, Figures 7A,B). However, SPC significantly improved mitochondrial state III respiration, the RCR, and the activities of NADH-OX, Cytc-OX, and SUC-OX in the diabetic rats after CoCl_2_ treatment, which was abolished by 2ME2 administration (Table 3).

**Table 3:** The changes in mitochondrial respiratory enzymatic activities under diabetic conditions.

	**NADH-OX**	**Cytc-OX**	**SUC-OX**
D	138.60 ± 7.89^∧^	43.06 ± 7.17^∧^	47.14 ± 6.62^∧^
I/R	121.12 ± 7.90	25.67 ± 5.18	35.17 ± 4.58
SPC	120.96 ± 8.84	26.57 ± 5.02	37.64 ± 4.68
COCl_2_	137.95 ± 7.68^∧^	42.24 ± 6.90^∧^	46.89 ± 6.56^∧^
COCl_2_+ SPC	151.74 ± 8.69^∧^^#^	52.85 ± 5.87^∧^^#^	59.55 ± 5.84^∧^^#^
COCl_2_+ 2ME2 + SPC	122.49 ± 7.68^&^	24.75 ± 4.23^&^	34.00 ± 4.53^&^
COCl_2_+ 2ME2	122.28 ± 7.53^&^	26.68 ± 4.80^&^	36.46 ± 4.10^&^

*Data are presented as the mean ± SEM (n = 6). NADH-OX: NADH oxidase; Cytc-OX: Cytochrome C oxidase; SUC-OX: succinate oxidase; D: diabetes; I/R: ischemia reperfusion; SPC: sevoflurane post-conditioning; CoCl_2_: Cobalt chloride; 2ME2: 2-Methoxyestradiol*.

∧ *P < 0.05 vs. I/R group*;

# *P < 0.05 vs. CoCl_2_ group*;

& *P < 0.01 vs. CoCl_2_ + SPC group*.

#### Expression of HIF-1α, VEGF and eNOS proteins

**Figure 8:**
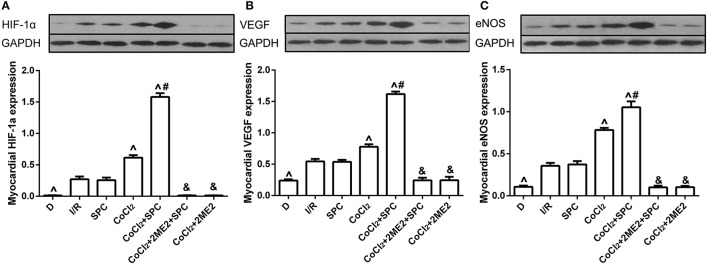
Under diabetic state, CoCl_2_ + SPC upregulated the HIF-1α VEGF, and eNOS expression. **(A)** HIF-1α expression. **(B)** VEGF expression. **(C)** eNOS expression (*n* = 3 /group). ^∧^*P* < 0.05 vs. I/R group; ^#^*P* <0.05 vs. CoCl_2_ group; and ^&^*P* < 0.01 vs. CoCl_2_ + SPC group.

Under diabetic conditions, SPC was unable to upregulate the protein expression of HIF-1α, VEGF and eNOS (*P* > 0.05 vs. I/R group). However, after CoCl_2_treatment, SPC significantly upregulated their expression levels in the diabetic rats, which was counteracted by administration of 2ME2 (Figures 8A–C).

#### NO content in myocardial tissue

SPC could not increase the NO content in diabetic myocardial tissue (*P* > 0.05 vs. I/R group), but could significantly enhance the NO content after CoCl_2_ treatment in diabetic rats. Nevertheless, 2ME2 abolished this SPC effect (*P* < 0.05, Table 4).

**Table 4:** The changes in myocardial NO and mitochondrial ROS under diabetic conditions.

	**ROS**	**NO**
D	5.93 ± 0.21^∧^	1.16 ± 0.07^∧^
I/R	12.78 ± 0.22	2.18 ± 0.09
SPC	12.55 ± 0.22	2.10 ± 0.05
COCl_2_	10.17 ± 0.22^∧^	3.08 ± 0.14^∧^
COCl_2_+ SPC	8.17 ± 0.12^∧^^#^	4.25 ± 0.11^∧^^#^
COCl_2_+ 2ME2 + SPC	12.39 ± 0.24^&^	1.26 ± 0.11^&^
COCl_2_+ 2ME2	12.57 ± 0.21^&^	1.22 ± 0.09^&^

*Data are presented as the mean ± SEM (n = 6). ROS: Reactive oxygen species; NO: Nitric oxide; D: diabetes; I/R: ischemia reperfusion; SPC: sevoflurane post-conditioning; CoCl_2_: Cobalt chloride; 2ME2: 2-Methoxyestradiol*.

∧ *P < 0.05 vs. I/R group*;

# *P < 0.05 vs. CoCl_2_ group*;

& *P < 0.01 vs. CoCl_2_ + SPC group*.

#### Mitochondria ROS level

SPC could not inhibit the mitochondrial ROS level after I/R damage in diabetic myocardium (*P* > 0.05 vs. I/R group), but could significantly upregulate the NO content after CoCl_2_ treatment in diabetic rats. However, 2ME2 abolished this effect of SPC (*P* < 0.05, Table 4).

### CoCl_2_ activates HIF-1α to restore the SPC-dependent protection of the diabetic myocardium, which is not associated with blood glucose levels

#### Cardiac function and myocardial infarction area

**Figure 9:**
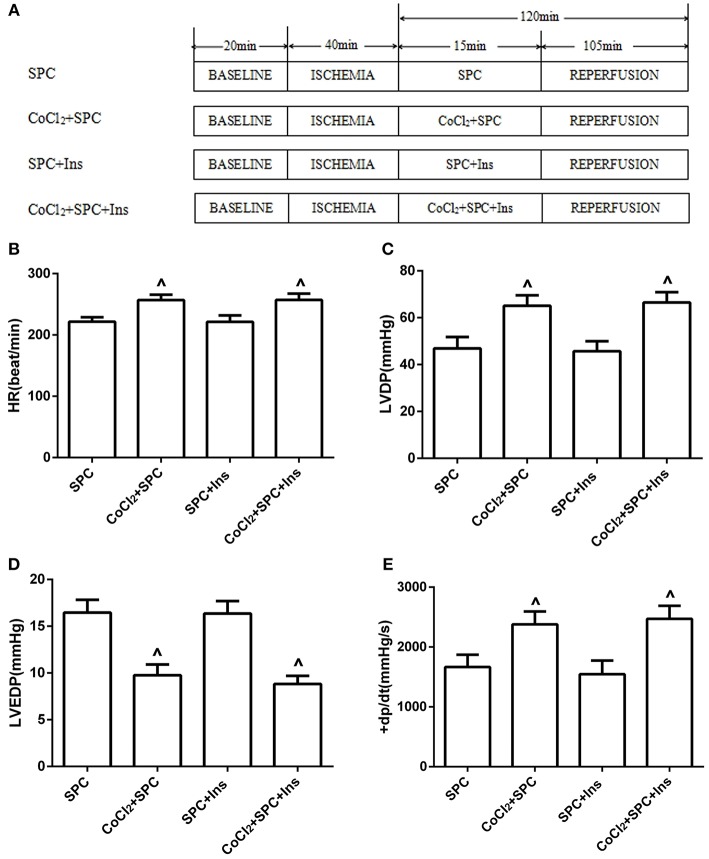
The schematic diagram of the experimental procedures. All hearts were subjected to 40 min whole heart ischemia, followed by reperfusion for 120 min. The SPC received 2.4% sevoflurane (1.0 MAC) treatment for 15 min, followed by reperfusion for 105 min. **(A)** The schematic diagram of diabetic rats treated by insulin. Under diabetic state, the improvement of cardiac function indicators was independent of blood glucose levels. **(B)** Heart rate. **(C)** Left ventricular developed pressure. **(D)** Left ventricular end-diastolic pressure. **(E)** Maximum rate of increase of LV pressure (*n* = 10 /group). ^∧^*P* < 0.05 vs. SPC group.

**Figure 10:**
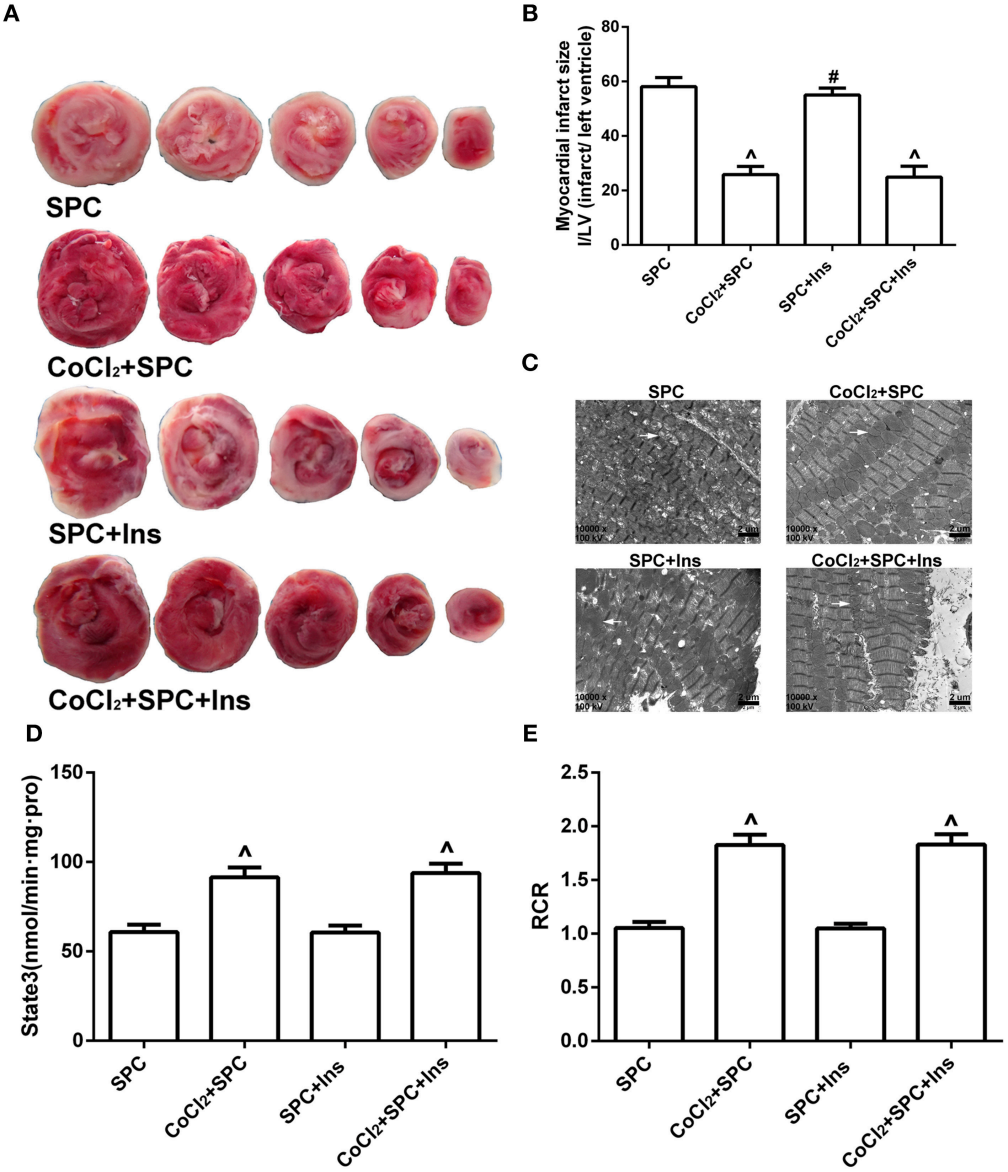
SPC reduced myocardial infarct area, and improved mitochondrial ultrastructure, mitochondrial respiratory function under diabetic conditions is not associated with blood glucose levels. **(A)** Myocardial infarct area. **(B)** The infarct size was expressed as infarct/ left ventricle (I/LV; *n* = 5 /group). **(C)** Myocardial ultrastructure, white arrows indicate intact mitochondria and damaged mitochondria. **(D)** Mitochondrial state III respiration. **(E)** Mitochondrial respiratory control rate (RCR; *n* = 10 /group). ^∧^*P* < 0.05 vs. SPC group, ^#^*P* < 0.05 vs. CoCl_2_ + SPC + Ins group.

To further examine the influence of blood glucose changes on SPC-dependent myocardial protection, insulin was used to manage the blood glucose levels. Our data revealed that there were no significant differences in the various hemodynamic parameters between the SPC group and the SPC + Ins group (*P* > 0.05; Figures 9B–E). Similarly, no differences were observed between the CoCl_2_+ SPC group and the CoCl_2_ + SPC + Ins group (*P* > 0.05). Moreover, insulin administration did not decrease the myocardial infarction area of the CoCl_2_+ SPC group (24.87 ± 4.11% vs. 25.91 ± 3.03%, *P* > 0.05, Figures 10A,B).

#### Mitochondrial ultrastructure

The SPC and SPC + Ins groups both exhibited extraordinary damage to the myocardial structure, including dissolved and even severed myofilaments. In addition, there was also apparent mitochondrial swelling, widened and ruptured ridges, and membrane gaps, as well as excessive expansion of the sarcoplasmic reticulum. In contrast, the mitochondrial morphology of the CoCl_2_ + SPC group and the CoCl_2_ + SPC + Ins group was intact with an orderly arrangement, although there was some swelling (Figure 10C).

#### Mitochondrial respiratory function and enzyme activity

After the blood glucose levels were decreased by insulin, the mitochondrial respiratory function of the CoCl_2_ + SPC group and the CoCl_2_ + SPC + Ins group was not affected (Figures 10D,E). Likewise, the administration of insulin did not significantly affect the mitochondrial activities of NADH-OX, Cytc-OX, and SUC-OX (Table 5).

**Table 5:** The influence of mitochondrial respiratory enzymatic activities by blood glucose levels.

	**NADH-OX**	**Cytc-OX**	**SUC-OX**
SPC	120.96 ± 8.84	26.57 ± 5.02	37.64 ± 4.68
COCl_2_+ SPC	151.74 ± 8.69^∧^	52.85 ± 5.87^∧^	59.55 ± 5.84^∧^
SPC + Ins	120.56 ± 9.12	23.91 ± 5.67	34.60 ± 5.70
COCl_2_+ SPC + Ins	149.96 ± 8.94^∧^	54.71 ± 5.61^∧^	59.06 ± 5.75^∧^

*Data are presented as the mean ± SEM (n = 6). NADH-OX: NADH oxidase; Cytc-OX: Cytochrome C oxidase; SUC-OX: succinate oxidase; SPC: sevoflurane post-conditioning; CoCl_2_: Cobalt chloride; Ins: Insulin*.

∧ *P < 0.05 vs. SPC group*.

## Discussion

This study demonstrated that under non-diabetic conditions, SPC-dependent myocardial protection is associated with the upregulation of both HIF-1α and its downstream mediators VEGF and eNOS. HIF-1α signaling is impaired under diabetic conditions, and this leads to the loss of SPC-dependent myocardial protection. Nevertheless, treatment with CoCl_2_ activated the damaged HIF-1α signaling, thereby restoring SPC-dependent myocardial protection under diabetic conditions. This effect was manifested by the upregulation of HIF-1α and its downstream mediators VEGF and eNOS, which improved NO, mitochondrial respiratory and enzymatic function and therefore decreased the infarction area and ROS, stabilized cardiac hemodynamics, and prevented damage to the mitochondrial ultrastructure. This SPC-dependent myocardial protection after activation of the HIF-1 pathway was independent of the blood glucose levels.

Previous studies have demonstrated that perioperative administration of the inhaled anesthetic sevoflurane has protective effects on the myocardium (Freiermuth et al., 2016; Wu et al., 2017). Under non-diabetic conditions, SPC can function in myocardial protection, similar to that during ischemic preconditioning or post-conditioning, which is a very promising and important means of coping with perioperative myocardial I/R injury (Riess et al., 2014; Zhang et al., 2014; Cao et al., 2015; Lemoine et al., 2016). Unfortunately, myocardial protection by SPC disappears in the context of diabetes (Drenger et al., 2011; Tai et al., 2012; Gao et al., 2016), the exact mechanism of which remains unclear. However, most of the relevant studies have concentrated on the PI3K-Akt, ERK1/2, and GSK-3β signaling pathways, while overlooking the key role of the endogenous HIF-1α signaling pathway. Under hypoxic stress, normal cells may activate multiple signaling pathways such as the PI3K-Akt pathway, the NO-PKG pathway, and the adenosine pathway to counteract ischemic cell injury, and most of these pathways are regulated by HIF-1 (Cadenas et al., 2010; Bento and Pereira, 2011; Xie et al., 2017). Hence, HIF-1α is considered the rescue head quarters for I/R injury (Eckle et al., 2008). Unfortunately, the HIF-1α signaling pathway is impaired under diabetic conditions. Therefore, it is of interest whether SPC, which confers myocardial protection under diabetic conditions, is pertinent to the damaged HIF-1α signaling pathway.

To verify the aforementioned hypothesis, we first examined whether the administration of SPC could increase the expression of HIF-1α, VEGF, and eNOS in healthy rats that suffered I/R injury. The results showed that SPC significantly elevated the protein expression levels of HIF-1α, VEGF, and eNOS, and the myocardial infarction area and ROS were therefore decreased. Importantly, the SPC-dependent myocardial protection was reversed by an HIF-1α-specific inhibitor. Next, we examined SPC-dependent myocardial protection in diabetic animals and found that compared with the control group (I/R), SPC treatment did not improve cardiac function, infarction area, mitochondrial respiratory function, or mitochondrial ultrastructure, which was in agreement with Tai et al. (2012). To exclude the possibility of interference from hyperglycemia, insulin was administered to maintain blood glucose at normal levels. However, this did not reinstitute the SPC-dependent myocardial protection under diabetic conditions (Drenger et al., 2011).

Previous studies have revealed that CoCl_2_ can activate the HIF-1α pathway, which is impaired under diabetic conditions, via a mechanism related to the inhibition of prolylhydroxylase (PHD; Xi et al., 2004; Nordquist et al., 2015; Koivunen et al., 2016). Our results revealed that after the activation of HIF-1α by CoCl_2_, SPC significantly upregulated the expression of HIF-1α, VEGF and eNOS, and thus improved the hemodynamic parameters (i.e., HR, LVDP, LVEDP, and +dp/dt_max_), mitochondrial respiratory function (i.e., state III and the RCR), and enzymatic activities (NADH-OX, Suc-OX, and Ctyc-OX) and reduced the myocardial infarction area. Furthermore, these protective effects were abolished after administration of the HIF-1α-specific inhibitor 2ME2.

Under the conditions of cardiac ischemia, the upregulation of HIF-1α can improve myocardial tolerance for acute I/R injury, which is attributed to the HIF-1α-mediated upregulation of the downstream component VEGF. As a consequence, microvascular generation and oxygen carrying capacity are augmented. Hence, VEGF, which is a hypoxia-sensitive downstream component of the HIF-1α signaling pathway, plays a crucial role in counteracting myocardial I/R injury. We reasoned that once the HIF-1α signaling pathway is activated by CoCl_2_, the SPC-induced upregulation of HIF-1α increases VEGF expression, which promotes neovascularization in the ischemic areas. Consequently, mitochondrial respiratory function and enzymatic activities are ameliorated, and the myocardial infarction area is decreased.

In addition, this study showed that the NO content was significantly increased in normal myocardium in the SPC group after I/R injury. We hypothesized that under conditions of ischemia and hypoxia, SPC could upregulate HIF-1α expression and directly induces eNOS synthesis, resulting in NO production. NO plays a role in maintaining vasodilation, inhibiting platelet aggregation and leukocyte adhesion on blood vessels. These effects suggest that NO produces protective effects during I/R injury. Previous studies have indicated that eNOS shows contradictory changes in diabetic myocardium, including increase (Stockklauser-Farber et al., 2000), decrease (Sridulyakul et al., 2003) or no change (El-Omar et al., 2003). In this study, we found that in diabetic rats, SPC, together with CoCl_2_, could upregulate eNOS expression, resulting in an increase in the NO content, suggesting that the change in eNOS and NO levels during diabetes is a process that accompanies changes in pathogenesis. However, when HIF-1α selective inhibitors were added, eNOS expression and NO content were significantly reduced. Therefore, it was hypothesized that the protective mechanisms of CoCl_2_ in combination with SPC in I/R injury in diabetic myocardium can result from the increase in downstream expression of VEGF and eNOS after activation of HIF-1α, inducing the release of NO (Kerkela et al., 2013). This can result in the promotion of endothelial proliferation and migration, resulting in neoangiogenesis, improving coronary artery circulation and myocardial function, increasing blood flow and oxygen supply to tissues. This would eventually improve respiratory function and enzyme activity in mitochondria, decreasing the area of myocardial infarction.

Mitochondria are important locations for oxidative phosphorylation, and the production of ATP depends on the integrity of the structure and function of mitochondria. When myocardial I/R injury occurs, the damage to mitochondria causes a decrease in the level of oxidative phosphorylation accompanied by production of large amounts of ROS, which in turn attack mitochondria to cause further damage (Inserte et al., 2012), and appropriate amounts of ROS have protective effects in the myocardium (Chen et al., 2008). However, ischemia and reperfusion results in the production of large amounts of ROS, which attack mitochondrial membranes. This results in lipid oxidation that damages cardiolipin and causes release of cytochrome C from the outer mitochondrial membrane, resulting in damage to the membrane structure (Kagan et al., 2009). This increases mitochondrial damage, resulting in defects in myocardial energy supply. In this study, we found that in non-diabetic myocardial I/R injury, SPC can improve the respiratory function and enzyme activity of mitochondria and maintain the normal process of oxidative phosphorylation in mitochondria, thereby providing energy to the ischemic myocardium. Furthermore, the SPC group showed a significantly lower ROS production rate compared with that in the I/R group and the area of myocardial infarction was decreased. This shows that SPC can inhibit ROS overproduction-caused I/R injury (Gong et al., 2012).

Interestingly, our previous study found that SPC could upregulate HIF-1 to improve mitochondrial respiratory function, and HIF-1 could decrease mitochondrial ROS, thus preventing damage to the myocardium (Hwang et al., 2015). Hence, we speculate that the mechanism maybe related to an increase in the expression of downstream VEGF after HIF-1α upregulation, which enhances the regeneration of ischemic capillaries. In addition, upregulation of HIF-1α can reduce ROS from mitochondrial sources (Hwang et al., 2015), avoiding mitochondrial damage caused by excessive ROS. CoCl_2_ can inhibit PHD to simulate a hypoxemic state, which stabilizes HIF-1α expression (Yuan et al., 2003). Under conditions of ischemia and hypoxia, HIF-1α upregulation can increase the expression of downstream VEGF and eNOS and improve respiratory function and enzyme activity in the mitochondria (Hwang et al., 2015), thereby promoting cell survival. In this study, we demonstrated that after CoCl_2_ activated the damaged HIF-1α signaling pathway during diabetes, SPC could stabilize and upregulate HIF-1α and increase the expression of downstream VEGF and eNOS, thereby improving the respiratory function and enzyme activity of the mitochondria and reducing the production of ROS, which plays a role in myocardial protection.

## Conclusions

This study demonstrates for the first time that CoCl_2_activates the HIF-1α signaling pathway, which restores SPC-dependent myocardial protection under diabetic conditions. Its underlying mechanism involves the SPC-induced upregulation of HIF-1α and its downstream mediators VEGF and eNOS, further resulting in an increase in NO content and decrease in ROS production, which leads to the stabilization of mitochondrial respiratory function and enzymatic activities.

## Author contributions

Conceived and designed the experiments: HZ, JW, and TY. Performed the experiments: JW, LY, JY, YM, HM, and PX. Analyzed the data: JW, LY, YY, HW, and PX. Wrote the paper: JW, PX, HZ, HW, and JW.

### Conflict of interest statement

The authors declare that the research was conducted in the absence of any commercial or financial relationships that could be construed as a potential conflict of interest.
